# Development and psychometric testing of a scale for assessing the psychological birth trauma

**DOI:** 10.3389/fpsyg.2023.1071336

**Published:** 2023-02-14

**Authors:** Ke Zhang, Meiliyang Wu, Tieying Zeng, Mengmei Yuan, Ye Chen, Lingyan Yang

**Affiliations:** ^1^Tongji Hospital, Tongji Medical College, Huazhong University of Science and Technology, Wuhan, China; ^2^School of Nursing, Tongji Medical College, Huazhong University of Science and Technology, Wuhan, China

**Keywords:** psychological birth trauma, women, reliability, validity, China, Birth Trauma Scale, BTS

## Abstract

**Background:**

Psychological birth trauma is widespread in postpartum women, and its harms are serious to mothers’ health. Existing tools rely on posttraumatic stress disorder to evaluate, which cannot effectively evaluate its connotation. The aim of this study was to develop a new instrument for use to comprehensively assess the psychological birth trauma level of women after birth and test the scale’s psychometric properties.

**Methods:**

The scale was developed and evaluated through item generation, expert consultation, pre-survey, and psychometric evaluation. A literature review, focus group, and individual deep semi-structured interviews were utilized to identify the scale items. The expert consultation evaluated the content validity. Psychometric testing was conducted in a convenience sample of 712 mothers within the first 72 h postpartum who were recruited from three hospitals in China.

**Results:**

The total Cronbach alpha coefficient of the scale was 0.874. Exploratory factor analysis supported that the final scale consisted of four dimensions and fifteen items. The explanatory variance of the four factors was 66.724%. The four dimensions are named “being neglected,” “out of control,” “physiological emotional response,” and “cognitive behavioral response.” The results of the confirmatory factor analysis showed that the fit indices were all at acceptable and good levels.

**Conclusion:**

The 15-item Birth Trauma Scale is a valid and reliable tool to evaluate the psychological trauma of mothers who experienced spontaneous childbirth. The scale is a maternal self-assessment scale that can help women understand their mental health. Healthcare providers can identify key populations and intervene with them.

## 1. Introduction

For most women, childbirth is usually a necessary process to become a mother, which has a significant impact on their bodies and mind. Physical injuries are usually treated promptly and effectively because of their impact on daily life. However, the psychological problems caused by childbirth are not obvious in a short period of time (Beck, 2015). People tend to ignore some early symptoms without seeking professional help (Byrne et al., 2017). It is not until the postpartum depression, or even PTSD, causes significant distress in life, that there is any concern.

As a precursor to many mental illnesses, psychological birth trauma (PBT) is a kind of trauma in the mind that occurs around the experience of childbirth. It is defined as the feeling of psychological trauma caused by spontaneous childbirth or the events that occurred during childbirth (Simpson and Catling, 2016). Its subsequent effects are quite significant. There is a report that every year on the anniversary, mothers still recall that painful birth experience that haunts their lives now (Beck, 2006). Some mothers may deliberately refuse to breastfeed because of PBT and completely alienate their babies (Beck and Watson, 2019). Some mothers even exhibit socially detached behavior, which is very detrimental to their subsequent physical and mental health (Murphy and Strong, 2018). In addition, PBT may also affect the mother’s partner, midwife, and others. Some fathers have developed some of the symptoms of PTSD because they witnessed the birth experience of the mother (Daniels et al., 2020). Some midwives have also been affected psychologically by being important medical personnel in the saving process and dealing with various complex delivery situations, and some have even resigned as a result (Beck et al., 2015). Based on the predicted role and the subsequent impact of PBT, we need to focus on this issue.

The reported incidence of PBT was between 9 and 44% (de Graaff et al., 2018). The lack of validated assessment tools for the specific assessment of PBT may have contributed to the wide range of its incidence. Some researchers defined psychological birth trauma as the subjective expression of mothers who consider the birth process traumatic (Watson et al., 2021). They defined psychological birth trauma as the subjective expression of mothers who consider the birth process traumatic. This approach may vary from person to person, and assessment results are unstable. A certain study used part of the scale for the diagnosis of PTSD as the evaluation standard (Olde et al., 2005). This reliance on PTSD diagnostic criteria for partial assessment of symptoms does not reflect the specificity of the event of childbirth. Scholars have developed a scale based on diagnostic criteria of PTSD to evaluate birth trauma (Ayers et al., 2018). It takes the event of delivery into account but focuses only on the assessment of symptoms associated with the postpartum period while ignoring the subjective maternal feelings.

When assessing psychological childbirth trauma, in addition to focusing on postpartum stress responses, it should also include some traumatic experiences during childbirth. Studies have shown that being ignored by an obstetrician or midwife during labor and delivery for one’s emotional needs can be perceived as invasive (Beck, 2004). Studies have also shown that the feeling of loss of control during labor is a direct cause of psychological birth trauma in women (Stankovic, 2017). For women, a few unexpected experiences during labor can make them define the birth experience as traumatic (Taghizadeh et al., 2013). Therefore, the assessment of birth trauma should also take into account the feelings or thoughts during the birth process. By combining the traumatic experience during labor with the postpartum stress response, the assessment will provide a more complete picture of the extent of birth trauma.

The study aims to develop a scale for evaluating the psychological trauma of childbirth by combining a review of the experience of childbirth and the stress response after childbirth. An assessment tool with good reliability and validity is important for identifying psychological birth trauma. Moreover, subsequent effective measures to address it will be very relevant to improve maternal mental health and advance the experience of natural childbirth.

## 2. Methods

### 2.1. Study design

A quantitative study was conducted to develop and evaluate the Birth Trauma Scale (BTS) for use after birth. There were three stages: The first stage was item generation to measure the level of psychological birth trauma for mothers. In the second phase, expert consultation was undertaken to form the initial version of the BTS. In the third phase, pre-survey was conducted to determine the questionnaire. The psychometric testing was carried out to item analysis and assess the construct validity and reliability of the newly developed BTS.

### 2.2. Data collection

#### 2.2.1. Item generation

A literature search was performed by electronic databases, including CINAHL, EMBASE, PsycARTICLES, PubMed, Web of Science, CNKI, and SinoMed. The search terms were selected relating to psychological birth trauma, including traumatic childbirth, birth trauma, and psychological trauma of birth. Moreover, we conducted a qualitative study on the traumatic experience of childbirth among Chinese women, which tended to find a detailed description of the traumatic experience of childbirth from their personal experiences. The relevant content has been published (Zhang et al., 2020). Based on the literature review and the results of our qualitative study, we found 71 items about psychological birth trauma. After consolidating and adjusting, a total of 33 items were generated by group discussion. They were divided into 8 dimensions, four for review of the intrapartum experience (being neglected, deprivation of dignity, out of control, and catastrophizing) and four for the postnatal stress response (physiological response, emotional response, cognitive response, and behavioral response).

#### 2.2.2. Expert consultation

Two-round expert consultations were conducted. The criteria for selecting the experts were as follows: (1) having more than 10 years of relevant work experience; (2) having a senior professional title or doctoral degree; (3) volunteering to participate in this study; (4) informed consent. Twenty-three experts participated in the expert consultation, whose areas of expertise included obstetricians, midwives, and care managers. These experts are 1 male and 22 female, including 11 graduate instructors. They were invited to evaluate the importance of every item and the correlation between items and dimensions. According to the suggestion of experts, we removed the items “I felt like I was dying.” (The item is overly colloquial in presentation and has an exaggerated tone), and “I cannot sleep when I think about childbirth.” (This item does not belong under the dimension it belongs to), and so on. The adding items were “I did not feel like I’m being treated fairly,” “When I think of childbirth, I feel increased pain in my wounds,” and so on. Meanwhile, we adjusted the expression of items (“I blamed my baby for the pain of childbirth.” was adjusted to “My labor pains came from the baby.”). The initial scale with 35 items was confirmed, which maintained 8 dimensions.

#### 2.2.3. Pre-survey

Fifteen mothers were invited to browse and fill in the questionnaire, including the basic information and the initial BTS. To ensure every participant could easily understand and smoothly finish the questionnaire, we modified the expression of several questions and added the explanation for the professional questions. We could know how long it roughly took mothers to participate in the study through the pre-survey.

#### 2.2.4. Psychometric evaluation

Convenience sampling was adopted to recruit research participants from three hospitals in China. One is a general hospital in Wuhan, and two are special hospitals for women and children in Wuhan and Taiyuan. The inclusion criteria included: (1) experienced spontaneous delivery including the failure condition of vaginal delivery; (2) within the first 72 h postpartum; (3) clear consciousness and good expression; (4) informed consent and voluntary participation in this study. The exclusion criteria included: (1) combined with infectious diseases, such as AIDS or syphilis; (2) complicated with severe heart, liver, kidney, or other organic lesions; (3) history of mental disorders. The main purpose of the data collection is to conduct a psychometric evaluation, in which the exploratory factor analysis and confirmatory factor analysis methods are applied. Factor analysis should satisfy a sample size of at least 5 to 10 times the number of items (Tinsley and Tinsley, 1987). The initial version of the BTS has 35 items. Considering 20% of invalid questionnaires, the sample size of this study was at least 438.

The mothers were recruited according to the criteria from November 2020 to March 2021. Data was collected by paper and electronic questionnaires in three hospitals in China. Paper questionnaires were collected by the researcher himself and members of the research team, visiting the maternity ward of the hospital and collecting them face-to-face. Participants were asked to sign a paper version of the informed consent form and then fill out a paper version of the questionnaire, which was collected on the spot. The electronic questionnaire was commissioned from the nurses of the participating hospitals. Participants were invited to enter the webpage by scanning the QR code, filling in the questionnaire, and submitting it successfully to retrieve this data. The completion time of data collection was 4 ~ 15 min for each participant. Informed consent was obtained from all participants.

### 2.3. Data analysis

The IBM SPSS Statistics version 26 was used to analyze the general data and perform exploratory factor analysis. The IBM SPSS Amos version 24 was used to perform confirmatory factor analysis.

#### 2.3.1. Content validity

The content validity of the scale was evaluated by using a Likert 4-level scoring method: 1 = irrelevant, 2 = weak relevant, 3 = relevant, 4 = strong relevant. The item-level content validity index (I-CVI) was calculated by the proportion of experts giving the item a relevance of 3 or 4 (Polit and Beck, 2006). The scale-level content validity index (S-CVI) was obtained by an averaging calculation method. After two rounds of expert consultation, the S-CVI of the BTS was 0.942, and the I-CVI was 0.870 ~ 1.000.

#### 2.3.2. Item analysis

The methods included the *t*-test method, correlation coefficient method, Cronbach’s *α* coefficient, exploratory factor analysis, and empirical method for the returned questionnaire to select items. The criteria for item deletion are as follows: (1) Independent sample t-test method. High group and low group (sorting the subjects according to the total score, 27% of individuals with the highest score formed the high group, and 27% of individuals with the lowest score formed the low group) scores are not statistically different. (2) Correlation coefficient method. The correlation coefficient between the item and the total score was below 0.40. (3) Cronbach’s alpha coefficient. If Cronbach’s alpha coefficient of the scale remarkably increased without the item. (4) Factor analysis. The factor load of every item was above 0.4. According to the above item deletion criteria, a tentative questionnaire with 15 items was formed.

#### 2.3.3. Construct validity

The evaluation of construct validity adopted exploratory factor analysis and confirmatory factor analysis. The effective 712 samples are randomly divided into two parts using a random number generator. One part (*n* = 356) was used to establish the model theory framework by exploratory factor analysis. The other part (*n* = 356) was used to verify the model structure by confirmatory factor analysis.

#### 2.3.4. Reliability

The reliability was evaluated by Cronbach’s alpha coefficient and correlation coefficient of the split-half reliability. We divided the items into two parts according to the odd and even numbers of the serial number and then calculated the correlation between the two parts (Streiner, 2003).

## 3. Results

### 3.1. General characteristics of participants

According to the inclusion and exclusion criteria, 720 mothers participated in the study. However, a total of 712 (98.89%) questionnaires were effectively taken back. The details of general information are shown in Table 1.

**Table 1:** General characteristics of participants (*n* = 712).

Characteristics	Total (*n* = 712)		Group 1 (*n* = 356)		Group 2 (*n* = 356)	
	Mean/*N*	SD/%	Mean/*N*	SD/%	Mean/*N*	SD/%
Age	30.0	3.7	30.1	3.6	29.9	3.8
Education						
High school or below	114	16.0	53	14.9	61	17.1
Undergraduation	505	70.9	258	72.5	247	69.4
Graduation	93	13.1	45	12.6	48	13.5
Place of living						
Urban	654	91.9	328	92.1	326	91.6
Rural	58	8.1	28	7.9	30	8.4
Medical insurance						
Yes	676	94.9	340	95.5	336	94.4
No	36	5.1	16	4.5	20	5.6
History of pregnancy						
Yes	225	31.6	115	32.3	110	30.9
No	487	68.4	241	67.7	246	69.1
Number of fetus						
Singleton	701	98.5	352	98.9	349	98.0
Twin	11	1.5	4	1.1	7	2.0
History of childbirth						
Primipara	491	69.0	240	67.4	251	70.5
Multipara	221	31.0	116	32.6	105	29.5
Episiotomy						
Yes	159	22.3	73	20.5	86	24.2
No	553	77.7	283	79.5	270	75.8
Postpartum hemorrhage						
Yes	16	2.2	8	2.2	8	2.2
No	696	97.8	348	97.8	348	97.8
Instrumental delivery						
Yes	10	1.4	6	1.7	4	1.1
No	702	98.6	350	98.3	352	98.9

### 3.2. Validity

#### 3.2.1. Exploratory factor analysis

Analysis of 356 questionnaires showed that the KMO value was 0.913. In Bartlett’s sphere test, *p* < 0.001, which indicated that it was suitable for factor analysis. Several methods are introduced in the item analysis part, the detailed results are shown in Table 2. Based on this result and exploratory factor analysis, as well as referring to expert opinions, 20 items were deleted, leaving 15 items. Finally, a total of 4 common factors with characteristic values greater than one were extracted. They were named “Being neglected,” “Out of control,” “Physiological emotional response,” and “Cognitive behavioral response.” The eigenvalues of the four factors are 2.951, 2.522, 2.499, and 2.036. The variance contribution rates of the four factors are 19.673, 16.815, 16.659, and 13.576%. The dimensions could explain 66.724% of the total variance. The result of the exploratory factor analysis is shown in Table 3.

**Table 2:** Statistic results of item analysis (*n* = 365).

Item	*t*	Correlation coefficient	Cronbach’s alpha if item deleted	Factor loading
1	8.314*	0.536*	0.943	0.608
2	7.571*	0.616*	0.943	0.724
3	8.045*	0.516*	0.943	0.573
4	7.418*	0.595*	0.943	0.696
5	6.916*	0.619*	0.943	0.679
6	7.228*	0.601*	0.943	0.716
7	6.451*	0.621*	0.943	0.743
8	7.317*	0.582*	0.943	0.645
9	11.067*	0.548*	0.943	0.471
10	12.920*	0.592*	0.943	0.537
11	10.682*	0.566*	0.943	0.519
12	10.859*	0.582*	0.943	0.501
13	7.956*	0.604*	0.943	0.584
14	7.141*	0.450*	0.944	0.406
15	11.712*	0.491*	0.945	0.374
16	17.635*	0.689*	0.942	0.636
17	17.366*	0.671*	0.942	0.631
18	15.254*	0.703*	0.942	0.690
19	14.354*	0.683*	0.942	0.663
20	15.305*	0.639*	0.943	0.571
21	16.257*	0.749*	0.941	0.710
22	21.606*	0.716*	0.942	0.633
23	13.646*	0.774*	0.941	0.757
24	18.567*	0.660*	0.942	0.575
25	10.271*	0.678*	0.942	0.657
26	7.545*	0.602*	0.943	0.607
27	7.115*	0.588*	0.943	0.610
28	7.362*	0.648*	0.943	0.670
29	14.485*	0.524*	0.945	0.405
30	17.382*	0.596*	0.943	0.489
31	11.178*	0.650*	0.942	0.625
32	10.931*	0.635*	0.942	0.615
33	7.079*	0.573*	0.943	0.607
34	6.507*	0.520*	0.944	0.543
35	6.524*	0.528*	0.944	0.526

*p < 0.001.

**Table 3:** Factor loading of each item of BTS.

Items	Being neglected	Out of control	Physiological emotional response	Cognitive behavioral response
I felt like no one could help me during childbirth.	0.752			
I felt like no one cared about me during childbirth.	0.801			
I felt like no one understood my feeling during childbirth.	0.757			
I felt like no one responded to my request during childbirth.	0.897			
I felt like I cannot have a baby successfully during childbirth.		0.548		
I cannot control my emotions during childbirth.		0.920		
I cannot control my behavior during childbirth.		0.798		
I get short of breath when I think of giving birth.			0.852	
I sweat when I think of giving birth.			0.879	
I get depressed when I think of giving birth.			0.612	
I get nervous when I think of giving birth.			0.579	
My labor pains came from the baby.				0.690
My labor pains came from the medical staff.				0.721
This birth experience alienated me from my husband.				0.768
This birth experience made me refuse to breastfeed.				0.704

#### 3.2.2. Confirmatory factor analysis

The other 356 questionnaires were analyzed to establish a model and modify the model through the covariance correction index (a two-way connection is established between the residuals with a significantly higher MI value). After the correction, the model has a better fitting index. The details are shown in Table 4.

**Table 4:** Results of confirmatory factor analysis (*n* = 356).

Fit index	*χ* ^2^	df	*χ*^2^/df	RMSEA	CFI	GFI	NFI	IFI	TLI	RMR
Model of BTS	247.273	83	2.979	0.075	0.951	0.916	0.929	0.951	0.938	0.066
Reference value			<5	<0.08	>0.90	>0.90	>0.90	>0.90	>0.90	<0.08

χ2, chi square; df, degree of freedom; RMSEA, root mean square error analysis; CFI, comparative fit ındex; GFI, goodness of fit ındex; NFI, normed fit index; IFI, ıncremental fit ındex; TLI, Turker–Lewis ındex; RMR, root mean square residual.

### 3.3. Reliability

The Cronbach’s alpha coefficient was calculated for the whole scale as well as for each dimension. The Cronbach’s alpha coefficient for the scale as a whole was 0.874. The Cronbach’s alpha coefficients for four dimensions ranged from 0.804 to 0.904. These values were within the acceptable threshold. The correlation coefficient of split-half reliability for the scale as a whole was 0.774. The correlation coefficient of split-half reliability for four dimensions ranged from 0.763 to 0.882.

## 4. Discussion

This study combined literature reviews, interviews with mothers who have experienced childbirth trauma, discussions with the research group, and the item pool with 8 dimensions and 33 items. Invited 23 experts familiar with the different professional fields (medical, nursing, health management) and rich in clinical experience to conduct 2 rounds of the expert consultation. Then the initial scale included 35 items. Based on the opinions of the letter, the first draft of the questionnaire is revised repeatedly. The expression is more concise and easier to understand, and then pre-test and large-sample tests are carried out according to the questionnaire development process to screen the questionnaire items and test their reliability and validity. The final BTS included 15 items. The questionnaire can be used for the trauma experience of the parturient during childbirth and the postpartum stress response to birth. The measurement content of the scale is more comprehensive and can be used as a clinical measurement tool for the psychological trauma of the parturient. Medical staff can judge the mental health of mothers through BTS, then provide targeted health education and guidance, improve the mental health of the mothers and prevent further psychological problems caused by the psychological birth trauma.

The final scale is 4 dimensions, two of which are the review of intrapartum experience and two are the postnatal stress response. The postpartum performance is mainly composed of the physiological stress response, emotional stress response, cognitive stress response, and behavioral stress response. It is also in line with the four general stress response assessment aspects.

The “being neglected” dimension consists of 4 items. It is defined as a woman’s perception that she is not being taken seriously by medical staff or family members during labor and delivery, or that she is not receiving adequate care and understanding due to a lack of communication. Previous studies have also confirmed that lack of care during labor and delivery, or lack of communication with midwives and clinicians, can make labor and delivery traumatic (Beck, 2004). Some women are also angry that their needs have been ignored (Abdollahpour and Motaghi, 2019).

The “out of control” dimension consists of three items. It is defined as the woman’s lack of control over the birth process and her loss of emotional and behavioral self-regulation. The mother’s own weakness and exhaustion make the overall situation out of her control (Abdollahpour and Motaghi, 2019). Some women cry and scream unconsciously because of the intensity of labor pain, and are completely unable to regulate their emotions or even control their own bodies (Zhang et al., 2020).

The “physiological emotional response” dimension consists of four items. It is defined as the woman’s neuroendocrine activity being affected when she recalls childbirth, and her body experiences a series of changes or negative emotional reactions to varying degrees. The negative emotions during childbirth leave a psychological shadow on the mother and she is unable to deal with her fears in the postpartum period (Taghizadeh et al., 2014). Especially for first-time mothers, childbirth as a traumatic event can bring negative feelings such as shame and low self-esteem (Iles and Pote, 2014).

The “cognitive behavioral response” dimension consists of four entries. It is defined as the postpartum experience of looking to others for the cause of the painful birth experience, or the behavioral manifestations such as avoidance and detachment. Postpartum perceptions of childbirth may include fatalism (i.e., the belief that it is one’s fate that these bad things happen) or blaming others for the trauma (Ayers, 2007). Memories of childbirth may also be altered, and memories may be reworked or simply avoided and distanced from the people involved outright. The persistence of birth trauma is also reflected in the mother’s inability to forget the bad memories of the process (Taghizadeh et al., 2014). The woman becomes sensitive in marital life, shows emotional indifference, and even refuses to have sex (Taghizadeh et al., 2013). The attitude toward one’s child is also more negative, and difficulties in mother-infant relations appear (Iles and Pote, 2014).

The reliability and validity of the scale are acceptable. In general, a Cronbach’s alpha coefficient greater than 0.8 indicates excellent internal consistency, between 0.6 and 0.8 indicates good, and less than 0.6 indicates poor (Spitzer et al., 1978). The results of the study showed that the questionnaire’s overall Cronbach’s *α* coefficient was 0.874, indicating that the questionnaire had good internal consistency and that all items were able to measure the same topic of maternal psychological trauma during childbirth. Cronbach’s alpha coefficients ranged from 0.804 to 0.904 for each dimension. In this study, content validity and structure validity were used to evaluate the validity of the scale. The content validity of each item and the total questionnaire were ≥0.78, indicating that the questionnaire items were in good agreement with the topic. Four factors were extracted by exploratory factor analysis. The eigenvalues of all factors were >1, and the cumulative contribution rate of the total variance was over 60%. They were basically consistent with the original item, suggesting that the items in the factors were typical representative items. In the confirmatory factor analysis, the smaller the value of *χ*^2^/df, the closer the RMSEA to 0, the less the RMR to 0.1, and the closer the GFI, NFI, CFI, TLI, and IFI to 1, indicating that the model fit is better (Tinsley and Tinsley, 1987). The confirmatory factor analysis results of this study showed that the model fits well.

This study has the following limitations. This study was based on a convenience sampling method and participants were recruited from the obstetrics and gynecology departments of tertiary care hospitals; future studies could evaluate this instrument at different levels of hospitals. Pregnant women who had planned cesarean sections were not included in this study. In addition, the scale was developed in a Chinese cultural context and may not be generalizable to other populations. The reliability of the scale could be tested in the future in more diverse contexts and populations.

## 5. Conclusion

The 15-item BTS is shown to be a valid and reliable tool to evaluate the psychological trauma of mothers who experienced spontaneous childbirth, which is considered in terms of four dimensions: “being neglected” (4 items), “out of control” (3 items), “physiological emotional response” (4 items), and “cognitive behavioral response” (4 items). The self-assessment BTS allows women to access their postpartum mental health status. In subsequent clinical applications, medical staff can identify women who score high on the BTS scale based on the results of the BTS assessment. For these key individuals, focused attention and psychological intervention can prevent the subsequent development of more severe psychological disorders.

## Data availability statement

The raw data supporting the conclusions of this article will be made available by the authors, without undue reservation.

## Ethics statement

The studies involving human participants were reviewed and approved by Ethics Committee of Tongji Hospital of Tongji Medical College of Huazhong University of Science and Technology. The patients/participants provided their written informed consent to participate in this study.

## Author contributions

KZ conceived, designed and performed the research, analyzed the data, and wrote the manuscript. MW conceived, designed and performed the research, and wrote the manuscript. TZ reviewed the drafts of the manuscript, monitoring the whole process of the study, and contributed to the technical or material support of the study. MY and YC performed the research, analyzed the data, and wrote the manuscript. LY performed the experiments and analyzed the data. All authors contributed to the article and approved the submitted version.

## Funding

This project was supported by the National Natural Science Foundation of China (No. 71974061).

## Conflict of interest

The authors declare that the research was conducted in the absence of any commercial or financial relationships that could be construed as a potential conflict of interest.

## Publisher’s note

All claims expressed in this article are solely those of the authors and do not necessarily represent those of their affiliated organizations, or those of the publisher, the editors and the reviewers. Any product that may be evaluated in this article, or claim that may be made by its manufacturer, is not guaranteed or endorsed by the publisher.
